# Short- and Long-Range Mechanical and Chemical Interphases Caused by Interaction of Boehmite (γ-AlOOH) with Anhydride-Cured Epoxy Resins

**DOI:** 10.3390/nano9060853

**Published:** 2019-06-04

**Authors:** Media Ghasem Zadeh Khorasani, Anna-Maria Elert, Vasile-Dan Hodoroaba, Leonardo Agudo Jácome, Korinna Altmann, Dorothee Silbernagl, Heinz Sturm

**Affiliations:** 1 Bundesanstalt für Materialforschung und -prüfung (BAM), D-12205 Berlin, Germany; anna-maria.elert@bam.de (A.-M.E.); Dan.Hodoroaba@bam.de (V.-D.H.); Leonardo.Agudo@bam.de (L.A.J.); Korinna.Altmann@bam.de (K.A.); dorothee.silbernagl@bam.de (D.S.); Heinz.Sturm@bam.de (H.S.); 2 Department of Polymer Materials and Technology, Technical University Berlin, D-10587 Berlin, Germany; 3 Department of Mechanical Engineering and Transport Systems, Technical University Berlin, D-10587 Berlin, Germany

**Keywords:** epoxy nanocomposites, boehmite, interphase, Intermodulation AFM, SKPM, AFM-IR

## Abstract

Understanding the interaction between boehmite and epoxy and the formation of their interphases with different mechanical and chemical structures is crucial to predict and optimize the properties of epoxy-boehmite nanocomposites. Probing the interfacial properties with atomic force microscopy (AFM)-based methods, especially particle-matrix long-range interactions, is challenging. This is due to size limitations of various analytical methods in resolving nanoparticles and their interphases, the overlap of interphases, and the effect of buried particles that prevent the accurate interphase property measurement. Here, we develop a layered model system in which the epoxy is cured in contact with a thin layer of hydrothermally synthesized boehmite. Different microscopy methods are employed to evaluate the interfacial properties. With intermodulation atomic force microscopy (ImAFM) and amplitude dependence force spectroscopy (ADFS), which contain information about stiffness, electrostatic, and van der Waals forces, a soft interphase was detected between the epoxy and boehmite. Surface potential maps obtained by scanning Kelvin probe microscopy (SKPM) revealed another interphase about one order of magnitude larger than the mechanical interphase. The AFM-infrared spectroscopy (AFM-IR) technique reveals that the soft interphase consists of unreacted curing agent. The long-range electrical interphase is attributed to the chemical alteration of the bulk epoxy and the formation of new absorption bands.

## 1. Introduction

Introducing boehmite nanoparticles (BNPs) as a nanofiller in polymer nanocomposites and the investigation of the associated property enhancements has been the focus of many recent studies [1,2,3,4,5,6]. The addition of BNPs to epoxy resins results in remarkable improvement in Young’s modulus, flexural modulus, thermal conductivity, compressive strength, dimensional stability, and fracture toughness [7,8,9,10,11,12,13,14]. Also, when the BNP-epoxy was used as a matrix in carbon-fiber reinforced composites, a remarkable enhancement of the fiber-matrix interphase was reported [15].

It is worth noting that the curing agent plays an important role in the properties of the nanocomposite since each curing agent can interact differently with boehmite nanoparticles. The different interaction between BNPs and epoxy components results in property variations, not only at the interface between polymer and filler but also the bulk properties of the cured polymer can vary significantly [16,17].

Chemical and physical interactions at the polymer–nanoparticle interface and the formation of an interphase with different properties than those of both filler and matrix phase has a major effect on property improvement in polymer nanocomposites. The formation of additional soft or hard phases as a result of particle–matrix interactions leads to new energy dissipation pathways and significant improvements in damage tolerance, crack resistance, and fracture toughness of the nanocomposite. Therefore, an in-depth understanding of interfacial characteristics at short and long distances from the interphase of nanoparticles is crucial. Numerical and empirical approaches show that strong bonding between a homogeneous polymer and nanoparticles generates a “mechanical interphase”, which is usually short-range in nature, and that the modulus gradient of the interphase does not exceed more than a few nanometers [18,19,20,21]. In a heterogenous polymer mixture consisting of resin and hardener, each molecule interacts with nanoparticles in different ways. Besides local distortions of the curing process due to confinement effects, chemical interactions between polymer and nanoparticles, as well as preferential adsorption of one component, can occur in a thermosetting matrix with nanofillers [22]. Based on functional groups on the surface of the filler, one compound is energetically preferred, and therefore one reactant is accumulated at the interphase. The preferential adsorption leads to stochiometric changes in the curing mixture, if only on a smaller scale. As a result, a “chemical interphase” is defined, which can considerably exceed the range of nanometers [23].

Several hypotheses regarding the interphase properties of epoxy-BNP nanocomposites have been proposed. Arlt et al. suggested that a mechanical interphase may form around BNPs, which is hypothesized to be a result of disturbed cross-linking around the particles [7]. This argument is indirect and is based on the topography of the surface after washing away the soft residues. Numerical studies of Fankhänel et al. showed that a soft interphase results from the high number of particle–matrix bonds [24]. The width of the interphase obtained from their simulations does not exceed more than a few tens of angstroms.

Atomic force microscopy (AFM) provides direct visualization and measurement of the interphase properties. Different AFM modes have been used to probe the mechanics of the interphase. AFM force-distance curves (FDC) offers a suitable approach for probing the interphase between thin films, where the vertical resolution is more important than the lateral resolution [25]. AFM in different modes, such as force modulation [26] or displacement modulation [27], has been used to probe the local stiffness of interphases of fiber-reinforced polymers. With the development of intermodulation AFM (ImAFM), it is possible to record a complete force curve at each image pixel with significantly reduced surface damage [28].

Recently, we showed that the analysis of ImAFM force curves enabled us to visualize the formation of the mechanical interphase as well as probing the van der Waals and dissipative forces, which indicated the short- and long-range alteration of bulk epoxy in the presence of BNPs [16]. However, a precise probing of mechanical interphases and obtaining enough data within the scale of such small nanoparticles (less than 20 nm) are still challenging, even with AFM approaches with a lateral resolution of a few tens of nanometers. Moreover, when probing the surface of a nanocomposite sample even with low volume fractions, long-range chemical interphases can overlap. Since the above-mentioned force measurement approaches are not sub-surface sensitive, the effect of buried particles under the surface can be overlooked in the measurements and result in wrong interpretation of long-range interphases and bulk properties. Therefore, in this study we aim to probe short- and long-range interfacial properties on a two-dimensional layered model sample with a controlled geometry. Having a layer of crystalline boehmite, approximately 1,000 nm thick, growing normal to the substrate plane, will result in a high-specific surface area and produce a localized interphase with a more pronounced mechanical and chemical property difference than boehmite and bulk epoxy. Thus, the effect of overlapping interphases and buried particles in the bulk polymer is minimized and measuring the thickness of affected areas becomes more feasible. Besides implementing ImAFM to study the mechanical interphases, we also show how scanning Kelvin probe microscopy (SKPM) and AFM-infrared spectroscopy (AFM-IR) can be used to detect and characterize the long-range effect of boehmite on the formation of chemically altered phases in the bulk epoxy.

## 2. Experimental

### 2.1. Materials and Sample Preparation

**Figure 1:**
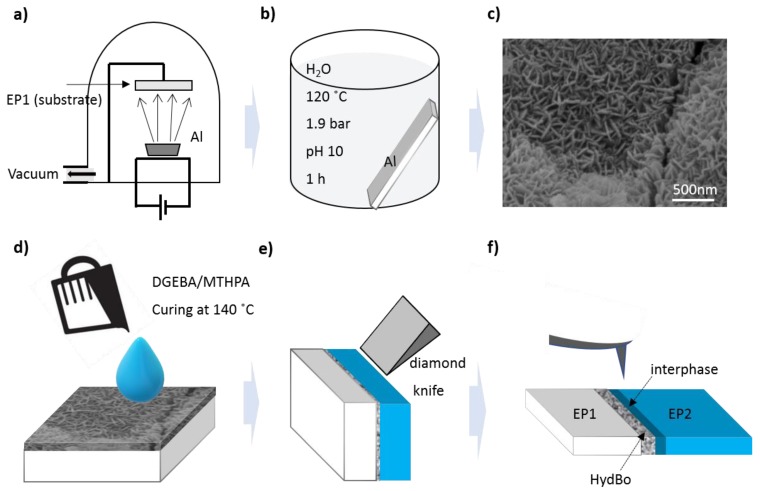
Schematic of the preparation of pre-cured epoxy substrate (EP1), hydrothermally synthesized boehmite (HydBo) and interactive epoxy surface (EP2) model sample. (**a**) Physical vapor deposition of aluminum on the epoxy substrate EP1, (**b**) Hydrothermal synthesis of the boehmite layer from the aluminum coating in distilled water, (**c**) Top view scanning electron microscope (SEM) image of the hydrothermally synthesized boehmite (HydBo), (**d**) Preparation of the interacting epoxy layer, (**e**) Ultramicrotomy of the cross-sectional surface of the layered structure. (**f**) Evaluation of the microtomed sample with atomic force microscopy (AFM)-based and SEM/EDS methods.

The sample preparation process is schematically shown in Figure 1. First, a 100 nm layer of aluminum was deposited on the surface of a pre-cured epoxy substrate (EP1, Figure 1a) using a physical vapor deposition (PVD) system (Edwards auto 306, Warley, UK).

The second step was the hydrothermal synthesis of boehmite (HydBo): the formation of boehmite by boiling distilled water on aluminum has been described elsewhere [29,30,31]. It was suggested that boehmite is produced via the reaction of aluminum ions migrating from the metal, reacting with hydroxyl ions and precipitating back onto the substrate [29]. More recent studies have demonstrated that the size and morphology of crystals can be controlled by variation of precursors, synthesis temperature, time, and pH value [32,33].

Here, we boiled the substrates with an aluminum coating with distilled water at 120 °C, under high pressure (1.9 bars). The pH value was adjusted to 10 by adding NH_3_ to the water solution. The solution containing the sample was transferred to a Teflon container, sealed, and heated for one hour. The sample was washed, dried at room temperature overnight, and further characterized by scanning electron microscope (SEM) (Figure 1c). As seen in Figure 1c, the outer region of boehmite consists of nanostructured (in the form of interconnected) lamellas interlocking in various directions in which the (100) planes are parallel to the substrate surface. A similar morphology of boehmite was reported when preparing boehmite coating by hydrolysis of AlN [34].

X-ray photoelectron spectroscopy (XPS) results show that the chemical composition of hydrothermally synthesized boehmite (HydBo) is in good agreement with commercially available BNP (for more details, see Appendix A).

In the next step, a polymer mixture consisting of resin, hardener, and accelerator was prepared, poured onto the surface of HydBo in a silicone mold, and cured to form the interactive epoxy surface (EP2, Figure 1d). The epoxy systems used in this study for both the substrate (EP1) and the interactive layer (EP2) are identical, consisting of Bisphenol-A-diglycidyl ether (DGEBA, Araldite® LY 556, Huntsman) cured with anhydride curing agent methyl tetrahydrophtalic acid anhydride (MTHPA, Aradur® HY 917, Huntsman) and accelerated by an amine, 1-methyl-imidazole (DY070, Huntsman). The mixing ratio of epoxy, hardener, and accelerator (by weight) is 100:90:1 parts, respectively. The mixtures were cured for 4 h at 80 °C to reach gelation and 4 hours at 140 °C for post-curing. EP1 has a different temperature history and curing conditions compared to EP2, since it was present during the evaporation of aluminum, the hydrothermal process and the curing of EP2. Thus, the two epoxies are not expected to exhibit identical properties. EP1 was used as the substrate to enhance the cutting procedure and easily access the cross-sectional surface for further measurements. The cross-sectional surface of the sample was further cut using an ultramicrotome (Leica Ultracut UCT) equipped with a diamond knife (DiATOME) (Figure 1e). For SEM and transmission electron microscopy (TEM) measurements, slices of approximately 100 nm in thickness were cut from the cross-sectional sample and were used with no extra conductive coating.

### 2.2. Characterization Methods for Short- and Long-Range Mechanical and Chemical Interphases

Different AFM-based methods were used to obtain physical, mechanical, and chemical properties of the material (Figure 1f). For high-resolution mechanical characterization of the surface, intermodulation AFM (ImAFM) was carried out. In this method, the cantilever is excited with two frequencies close to the resonance of the cantilever *ƒ*_res_. Here, we chose frequencies 0.5 kHz above and below the frequency of the first flexural eigenmode of the cantilever (*ƒ*_1_ = *ƒ*_res_ − 0.5 kHz and *ƒ*_2_ = *ƒ*_res_ + 0.5 kHz). The resulting free oscillation of the cantilever is in the form of a beating wave. This motion is distorted by the nonlinear tip–sample interaction when the tip comes close to the surface. As a result, additional frequency components in the cantilever motion appear, which are called intermodulation products (IMPs). During the scan, amplitudes and phases of IMPs are measured with a multi-frequency lock-in amplifier. At each scan pixel, the oscillation of the cantilever is recorded through a cycle that takes less than a few milliseconds. Each cycle starts from low amplitudes, reaches a maximum, and then decreases back to zero. At the beginning of the oscillation cycle, the amplitude is low, therefore, there is no tip–surface interaction (the tip does not sense the sample yet). When the amplitude increases, the tip first starts sensing the electrostatic forces of the sample, followed by the attractive van der Waals forces. Further increasing the amplitude results in the contact of the tip with the sample surface and further penetration into the surface. In this region the tip experiences a net repulsive force that gives information about the surface stiffness. The force obtained at this stage is a function of amplitude, thus, it cannot be treated directly as conventional force-distance curves. Amplitude-dependence force spectroscopy (ADFS) uses the inverse Abel transform to convert the curve to a traditional force-distance curve [28,35]. In the latter, the slope of the curve in the repulsive regime gives a quantitative measure of the stiffness and the forces in the attractive regime are related to electrostatic and van der Waals forces.

The second AFM-based method used in this study is scanning Kelvin probe microscopy (SKPM). In this method, a capacitor plate forms between the tip and the surface, where their potential difference is measured with air as the dielectric in between. Surface potential mainly depends on the difference between the work functions of the tip and the sample. SKPM provides a high-lateral resolution map of surface potential which can be used to probe heterogeneous phases with different structural and compositional properties. The cantilever is excited electrostatically at its resonance frequency by applying an AC signal. Due to the potential difference between the tip and surface, the cantilever begins to oscillate mechanically. By applying a bias voltage to the cantilever, the oscillation is cancelled out. This bias voltage is then collected, from which the surface potential is calculated. The corresponding equations and technical considerations are described in detail elsewhere [36]. The measurements are carried out as a dual pass approach in which two scans per line were performed. The first scan obtains the topography, which is then used in the second scan to maintain a certain distance from the surface while measuring the potential.

For ImAFM and SKPM measurements in this study, a MFP3D microscope (Asylum Research, Santa Barbara, CA, USA) was implemented. The gold-coated silicon probes were provided by Mikromasch (Wetzlar, Germany), with a radius lower than 20 nm and resonance frequency *ƒ*_res_ = 185 kHz. During all SKPM measurements, the nap height was chosen to be 50 nm as the suitable height according to the topographic features of the surface. The measurements were carried out in air, at room temperature, using the first eigenmode frequency.

AFM-based infrared spectroscopy (AFM-IR) is a hybrid technique where chemical characterization provided by infrared (IR) spectroscopy can be obtained at the spatial resolution of AFM [37,38,39,40]. This is achieved by using the gold-coated tip of AFM to locally detect thermal expansion of a sample resulting from local absorption of IR radiation. Therefore, the AFM cantilever acts in this method as the IR detector, allowing the AFM-IR technique to overcome the spatial resolution limits of conventional IR microscopy. A tunable infrared laser is applied as the source of IR radiation. The IR light is focused onto a sample region in the proximity of the AFM tip. If the laser is set to a wavelength that corresponds to an absorption wavelength of the sample, the sample sees a rapid thermal expansion by absorbing the light. The resulting expansion causes a force impulse on the tip of the cantilever, inducing an oscillation of the cantilever, whose amplitude can then be detected. By tuning the repetition rate of the laser to match one of the contact mode resonance frequencies of the AFM cantilever, more sensitive and faster measurements are possible. By measuring the AFM probe oscillation amplitude response to IR absorption as a function of wavelength, it is possible to readily create IR absorption spectra of nanoscale regions of the sample surface.

The AFM-IR data were obtained using a NanoIR2s (Bruker/Anasys Instruments) coupled with a multichip quantum cascade laser (QCL) source (MIRcat, Daylight Solutions; tunable repetition rate range of 0–500 kHz; spectral resolution of 0.1 cm^−1^) covering the range from 900 cm^−1^ to 1900 cm^−1^. An Au-coated silicon probe (tapping AFM-IR cantilever, Anasys Instruments-spring constant 1–3 nN·m^−1^) was employed.

The scanning electron microscope (SEM) was of type Zeiss Supra 40 (Zeiss, Oberkochen, Germany) being equipped with a Schottky field emitter and a high-resolution InLens detector for the secondary electrons. Additionally, the SEM was also operated in the STEM-in-SEM (or T-SEM) mode, by using a dedicated, transmission sample holder which uses typical TEM grids of 3 mm diameter. The sample was prepared as a microtome-slice (see Figure 1) on a carbon TEM grid without any additional coating. Various beam voltages (3, 5, 10, and 20 kV) were applied in order to catch both the best surface morphology and reasonable excitation of the X-ray lines considered for Energy Dispersive X-ray Spectroscopy (EDS) analysis (line scans and hyperspectral maps) across the layered structure. The EDS system used was of type Ultra Dry SDD EDS (Silicon drift detector energy dispersive X-ray spectrometer) from Thermo Fisher Scientific (Waltham, MA, USA) with a nominal active area of detector of 100 mm^2^. More details on the dedicated arrangement SEM/STEM-in-SEM/EDS can be found in [41].

For X-ray photoelectron spectroscopy (XPS) measurements, a Sage 100 (SPECS, Berlin, Germany) was used. The angle between the X-ray source and the analyzer was 54.9°. The axis of the analyzer forms an angle of 18°, with the sample surface normal. The samples were analyzed with non-monochromatic Al Kα radiation at a pressure of less than 3 × 10^−7^ mbar. The measurement area was 1 × 3 mm^2^. The spectra were taken at a current of 18 mA, and 10 kV and 20 eV pass energy in fixed analyzer transmission mode. The results are a mean value of two measurements. The software CasaXPS (Version 2.3.17PR1.1, Casa Software Ltd, Devon, Teignmouth, United Kingdom) was used to fit recorded signals.

## 3. Results and Discussion

### 3.1. Morphology of the Interfacial Region

**Figure 2:**
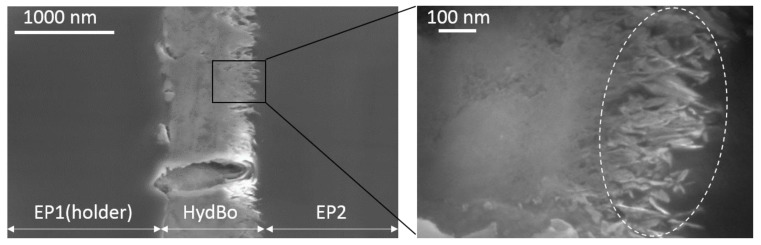
3 kV SEM micrograph of the cross-sectional surface of EP1/HydBo/EP2 layered structure prepared as microtome slice on a hollow transmission electron microscopy (TEM) grid (without additional coating).

The microtomed cross-sectional surface of the layered sample was first analyzed using scanning electron microscopy (SEM) and is presented in Figure 2. The average thickness of the HydBo layer was approximately 1 µm. The interface between HydBo and epoxy was flatter on the left side (i.e., to EP1) of HydBo. Due to the morphology of the crystals in the outer region, no well-defined border between EP2 and HydBo was observed. As seen in the inset image of Figure 2, in the outer region of the boehmite layer towards EP2 (right side) the crystal growth orientation was rather perpendicular to the plane of the substrate (EP1). There, boehmite crystals showed thin nanoplatelets and needle-like morphology, approximately 100nm in length. This morphology was also confirmed by transmission electron microscopy (see Appendix B). Due to the high-surface area of such nanostructures in the outer region of the layer, a pronounced interaction between boehmite and epoxy within this region was expected (marked with a dashed oval in Figure 2). Thus, this board region is the area of interest in further AFM measurements. In the following, we analyze the mechanical and physical properties of epoxy (EP2), preferentially in this interfacial region.

### 3.2. ImAFM Analysis

**Figure 3:**
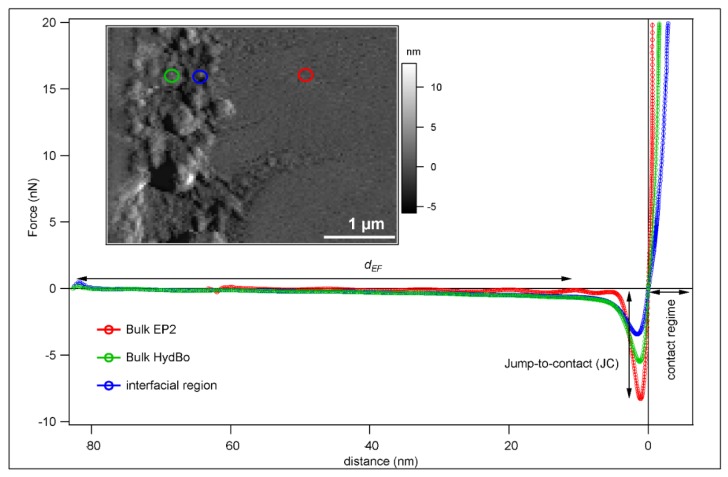
Averaged force-distance curves from amplitude dependence force spectroscopy (ADFS) measurements on point locations shown in the inset image (topography). See text for details.

The interfacial region described above was measured by means of ImAFM, as shown in Figure 3. The inset image of Figure 3 shows the AFM tapping mode topography of the cross-sectional surface of the sample. The topography image is consistent with the SEM micrograph in which the morphology of the HydBo layer is distinguishable from the epoxy. By implementing ImAFM, ADFS force curves are obtained for each pixel of the scanned area. Figure 3 presents the average curves from different regions of the scanned area including bulk EP2 (red), bulk HydBo (green), and the interfacial region (blue). It is observed that these curves have different characteristics in both attractive and repulsive regimes. As mentioned before, the repulsive regime occurs when the tip comes in contact with the sample surface due to the applied force (distance < 0, far right in Figure 3), and the slope of the curve in this region represents the stiffness of the sample. In the attractive regime, the tip experiences net attractive forces of a different nature, mainly governed by electrostatic and the van der Waals forces (distance > 0). The electrostatic forces can be originated by either the dissociation of the surface groups or by the absorption of ions onto the surface [42]. The influence of electrostatic forces is of a long-range nature and is observable in tip-sample separation distances above 20 nm, whereas van der Waals forces affect the tip in immediate distances to the surface [43]. In the vicinity of the surface, due to the van der Waals forces, the tip experiences the jump-to-contact [44]. Here, we use the force offset at jump-to-contact, which is herein named *F_JC_*, to map the van der Waals forces. The distance at which the slow zero-force offset starts, named *d_EF_*, is here used to map the intensity of electrostatic forces and the distribution of surface charges of the sample. Since the forces in the attractive regime are independent of the mechanical properties of the sample, the maps of electrostatic and van der Waals forces can be used as complementary channels of information to observe material contrast and to precisely distinguish the stiffness of organic and inorganic phases in the composite, as well as locating the mechanical interphase.

**Figure 4:**
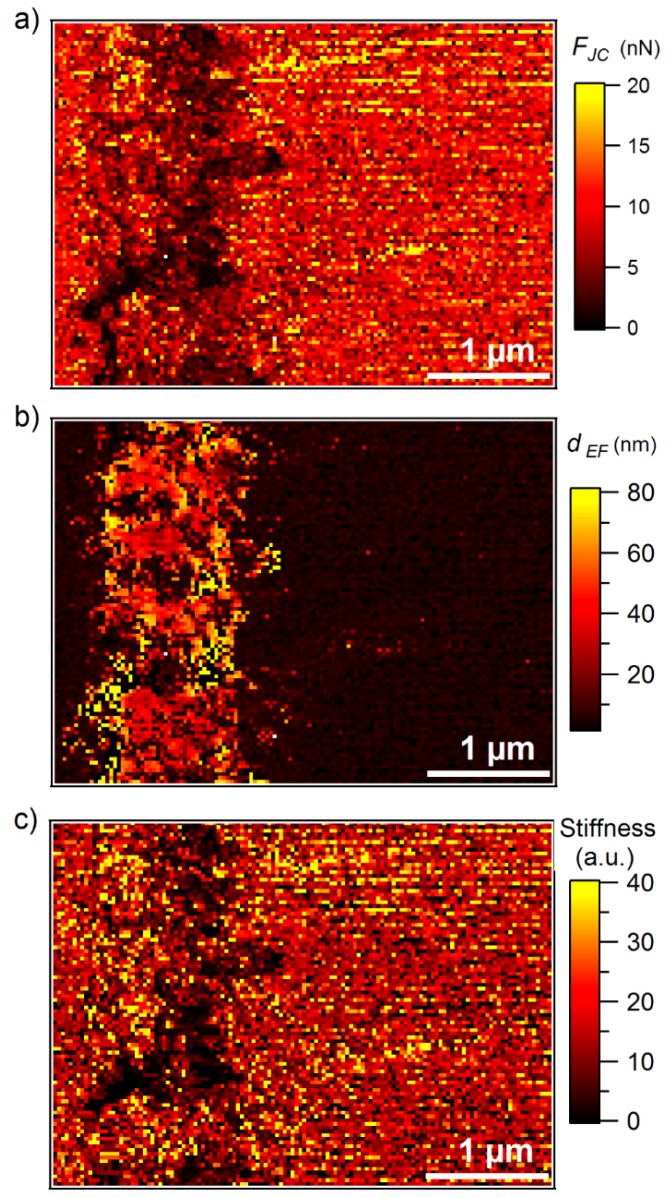
Maps of (**a**) attractive force (jump-to-contact height), (**b**) attractive force (electrostatic force), and (**c**) stiffness of the cross-section of EP1(left)/HydBo/EP2 (right) model sample.

Figure 4a,b show the maps of *F_JC_* and *d_EF_*, respectively. The map of stiffness, which is obtained by linear fit of the curve in the repulsive regime, is presented in Figure 4c. The map of *d_EF_* shows the material contrast between the bulk epoxy and the bulk HydBo layer. In the map of *F_JC_*, the interfacial region appears more pronounced, whereas the bulk HydBo and bulk epoxy do not differ significantly.

The stiffness map (Figure 4c) shows a similar contrast to the *F_JC_* map (Figure 4a), where the interfacial region has a very low stiffness compared to the bulk HydBo and bulk epoxy. There is no significant stiffness contrast between the bulk epoxy and bulk HydBo. This is in agreement with previous findings, where the stiffness values of cured epoxy and boehmite in nanocomposites did not show a remarkable difference [16]. The soft area at the interfacial region, which is approximately 100–200 nm thick, coincides with the location of the HydBo’s outer shell (nanoplatelets, described in Figure 2). These results show that where structures of boehmite with a high-surface area are exposed to a curing epoxy, a soft interphase is formed as a result of the interaction between epoxy components and boehmite surface. It is worth noting that additional studies of the mechanical properties of bulk epoxy (EP2) with the AFM force-distance curves (FDC) approach did not show any long-range (over 1 µm) stiffness gradient in the bulk epoxy (see Appendix C).

The formation of the soft interphase around BNPs in the epoxy matrix with approximately 50 nm thickness was previously reported [16]. It was hypothesized to be due to either disturbed cross-linking, confinement effects, or to preferential absorption of an epoxy component (either DGEBA or anhydride hardener), leading to a local phase segregation in the vicinity of the boehmite surface [16]. In Section 3.5, the AFM-IR measurements provide information about the chemical composition of the soft interphase.

### 3.3. Surface Potential Measurements

**Figure 5:**
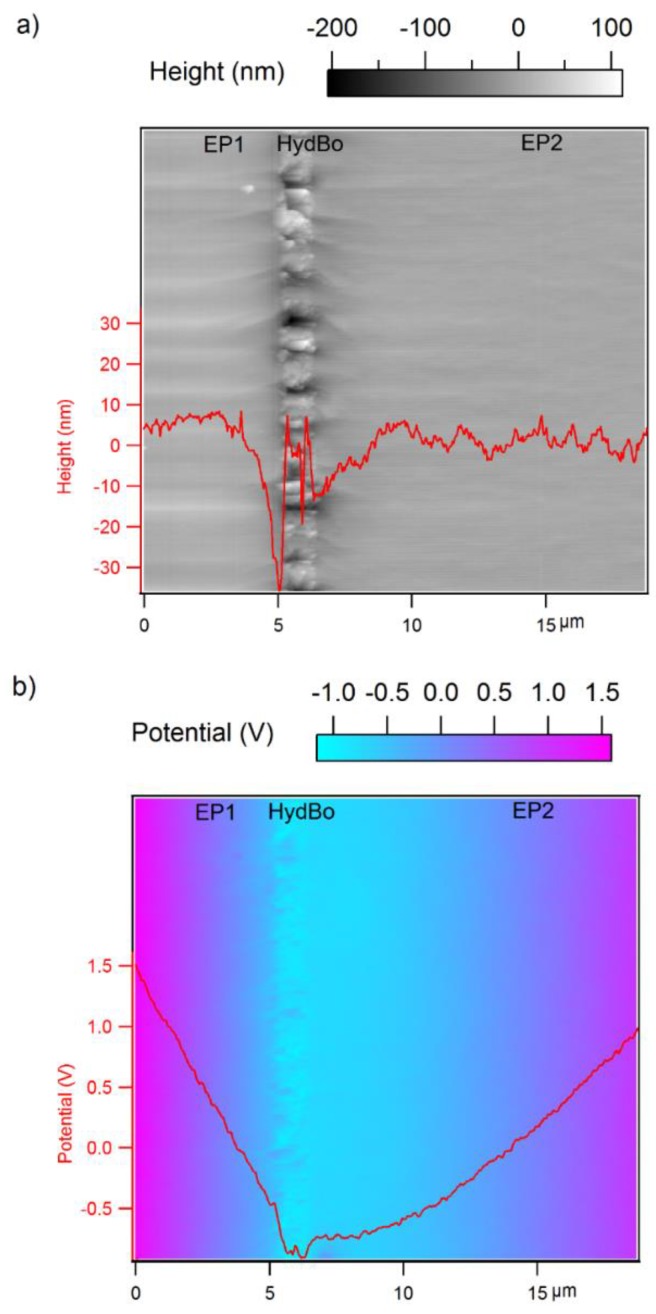
Maps of (**a**) topography and (**b**) surface potential of the cross-section of EP1/HydBo/EP2 model sample. The line profiles are presented in red.

Although bulk epoxy and bulk HydBo do not show a clear contrast in stiffness, their surface charges do show a remarkable contrast, as observed previously in the *d_EF_* map. For a better evaluation of the electrical properties, the sample was examined with SKPM. A larger scanned area (20 µm × 20 µm) enabled us to visualize the long-range effects, as seen in Figure 5. In the topography image (Figure 5a), the morphology of the HydBo layer is distinguished from the epoxy layers. In the potential map (Figure 5b), in addition to the potential difference between HydBo and bulk epoxy, a considerable potential gradient is also present, whereas on the EP2 side it extends over a wide region.

It was previously reported that in epoxy-boehmite nanocomposites, epoxy exhibits higher potential values than boehmite [16]. The difference in the width of potential gradient toward EP1 and EP2 is due to their different preparation histories. Due to the diffusion of aluminum through the surface of EP1 during the thermal deposition and further possible chemical bonding with the epoxy, a gradient in potential was expected at the interface of EP1 and HydBo. However, the gradient between HydBo and EP2 was five times broader than that of EP1. Such a long-range interaction was unexpected, mainly since no long-range mechanical interphase was observed in the force-distance curve measurements on the similar scanned area and the soft interphase around it was only formed around the outer shell of HydBo, with a less than 1 µm thickness. Therefore, we hypothesized that the nature of such a long-range potential gradient obeys chemical alterations in the bulk epoxy. This could be either the result of diffusion of HydBo nanostructures far into the bulk EP2 during the preparation/curing, or the alteration of the curing chemistry of epoxy due to the preferential absorption of epoxy components. These two hypotheses are further studied by elemental analysis using SEM-EDS and chemical analysis using AFM-IR, respectively.

### 3.4. Elemental Analysis via EDS (Energy Dispersive X-Ray Spectroscopy)

**Figure 6:**
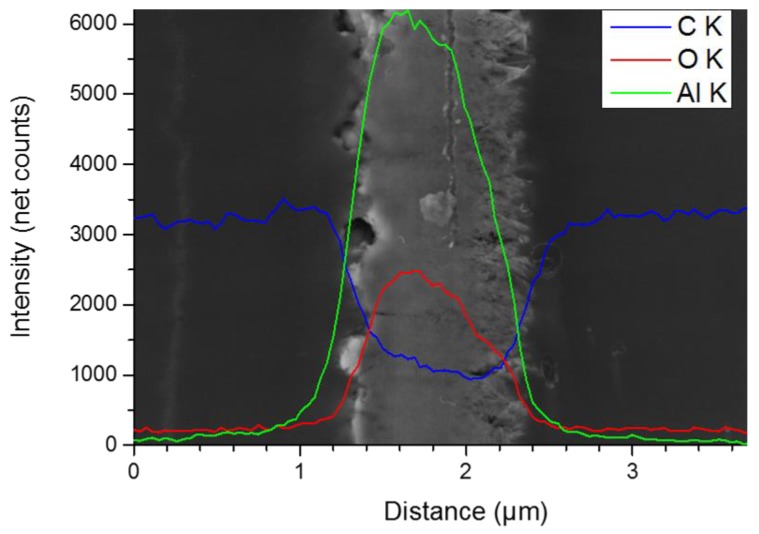
3 kV SEM micrograph of the cross-section of EP1 (left)/HydBo/EP2 (right) and a 20 kV Energy Dispersive X-ray Spectroscopy (EDS) linescan with C K, O K, and AL K signals as net intensities (after spectral background subtraction). The EDS linescan has been extracted from a 256 × 196 pixel hypermap (Appendix D) over the imaged area and after summing all of the net X-ray intensities along the direction parallel with the HydBo layer in order to get good counting statistics under gentle excitation conditions.

A representative SEM image of the cross-sectional surface of the EP1/HydBo/EP2 system, together with an EDS linescan with Al K, C K, and O K X-ray line intensities, are shown in Figure 6. An EDS hypermap (containing an EDS spectrum in each pixel) has been acquired over the area imaged in Figure 6 as an alternative to a conventional linescan (see Appendix D), in order to avoid beam damage due to long acquisition times for an analysis point. From the 256 × 196 pixels EDS hypermap acquired for 5 min, after 67 frames with only 4.4 s/frame, the EDS linescan shown in Figure 6 was extracted by summing the net X-ray intensities (after spectral background subtraction) along the direction parallel to the HydBo layer. Thus, good counting statistics were attained at gentle conditions (i.e., at high scan speed of the electron beam). It should be noted that the preparation of the thin, electron-transparent slice by microtomy resulted in a reduction of the spatial resolution of EDS from the conventional micrometer range, down to below 100 nm [41]. There was a clear asymmetry in the signals of Al, O, and C at the interphases of EP1 and EP2 with the HydBo layer. This is due to the different morphology of the HydBo layer, as observed in Figure 2. At the left side of the HydBo layer, in which the boehmite shows a more compact morphology, the intensity of the Al and O signals were higher (and the edge became sharper) than on the frayed right side, which interacts with EP2. Furthermore, on the right side of HydBo, where the long-range (~10 µm) potential gradient was observed (see Figure 5), the intensity of the Al signal dropped immediately at the interphase of EP2 and went to zero, approximately 1 µm away from the HydBo layer. This demonstrates that no significant long-range diffusion of boehmite into the bulk of the epoxy material was observed and thus this hypothesis does not explain the formation of the long-range potential gradient.

It is worth noting that the net intensity of the C K line inside the HydBo layer is not zero. This could be explained by the diffusion of epoxy inside the HydBo volume during the preparation and curing. However, a small contribution of carbon from surrounding epoxies (EP1 or EP2) might be co-excited by the X-rays generated in the not-perfectly-compact HydBo layer. The non-zero carbon signal in the HydBo layer has also been observed in other EDS linescans measured at different locations over the HydBo layer in the conventional linescan mode (point-by-point). It is reasonable to assume that EP2 components are absorbed physically or interact chemically with the surface of HydBo with its inherent roughness of at least 100 nm (Figure 2). Additionally, EP2 components penetrate through the porous surface structure of the HydBo layer. If this interaction is selective, meaning that one component (resin or hardener) is more likely to interact with the HydBo layer, a long-range chemical alteration of the bulk EP2 occurs consequently, which can result in the long-range potential gradient in EP2. Since a direct correlation between the carbon signal to the diffusion of epoxy into the HydBo layer is not conclusive from the EDS measurements, we further examined this hypothesis by investigating the local chemical structure of the epoxy using AFM-IR.

### 3.5. AFM-IR Analysis

Prior to AFM-IR, attenuated total reflectance infrared (ATR-IR) spectroscopy on pure DGEBA, MTHPA, HydBo and the cured epoxy were carried out (see Appendix E). The carbonyl group usually shows a strong intensity of absorption in the infrared spectrum C=O, which makes it useful to identify the anhydride component. The absorbance peaks at 1779 cm^−1^ and 1860 cm^−1^ are attributed to the symmetric and asymmetric stretch of C=O, respectively. Depending on the intra- and inter-molecular factors, these peaks can show deviations. Inter-molecular hydrogen-bridging between C=O and an external component can result in a slight decrease of absorption frequency. The curing of epoxy with anhydride results in the formation of an ester band at 1739 cm^−1^ and the disappearance of the asymmetric stretch of carbonyl [45]. This information is further used to interpret the AFM-IR results.

**Figure 7:**
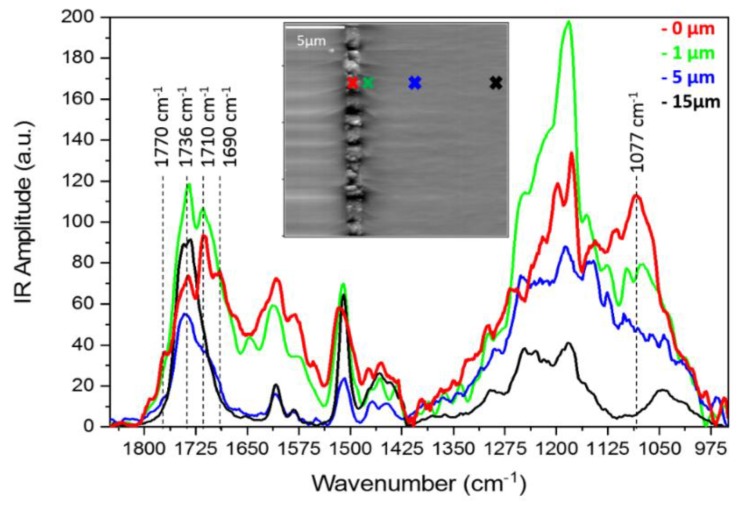
AFM-infrared spectroscopy (AFM-IR) spectra from point measurements shown in the inset AFM topography image of the cross-sectional surface of EP1/HydBo/EP2 model sample.

Local IR signals were collected from the cross-sectional surface of EP1/HydBo/EP2 samples at different distances (0, 1, 5, and 15 µm) from the HydBo layer. The spectra taken from the points with a similar distance to HydBo were averaged and are plotted in Figure 7. The absorbance at 1077 cm^−1^ is a typical boehmite band which is not overlapped with the absorbance of epoxy and thus, in our experiment it was used to identify the existence of boehmite [46]. It was observed that this absorption peak was not only present in the HydBo layer as expected, but also in the epoxy domain up to 1 µm away from the HydBo, and no absorption was observed at further distances (5 µm and 15 µm). This confirms that the diffusion of particles in the bulk polymer is only limited to short distances, as is also observed with EDS, where the aluminum signal was only pronounced up to 1 µm away from HydBo so that no boehmite was detected in the bulk EP2.

Besides the ester band at 1736 cm^−1^, which associates to the curing of epoxy with anhydride, an additional shoulder at 1770 cm^−1^ in the spectrum taken from the outer region of HydBo (0 µm) indicates the existence of unreacted anhydride absorbed by the HydBo layer. This peak gradually decreases with increasing distance from the interface and disappears completely at distances larger than 15 µm. The latter is the same distance where the potential gradient was observed in Figure 5. The presence of unreacted anhydride in the vicinity of HydBo indicates that the resin-hardener ratio is locally imbalanced and consequently, the curing and chemical properties of the epoxy network were also affected at long distances (up to 15 µm) from the interface. The absorbance bands at 1510 cm^−1^ and 1,608 cm^−1^ associated to the presence of the aromatic rings of DGEBA, which exists in all spectra including the spectrum obtained from the outer region of the HydBo layer (0 µm). Hence, DGEBA was also present within the HydBo layer. Since all the characteristic absorption bands related to oxirane (831, 915, and 3057 cm^−1^) were either overlapped with absorption of other species or out of the frequency limitation of AFM-IR, it was not possible to distinguish if the DGEBA component in these spectra was unreacted or part of a polymer chain.

**Figure A8:**
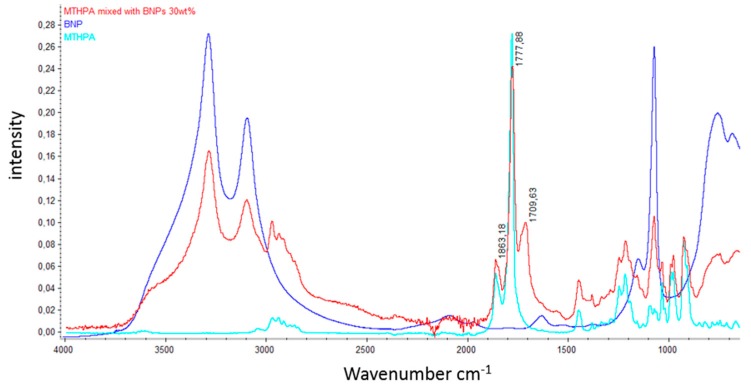
Room temperature ATR-IR spectra of BNPs, anhydride hardener and the masterbatch mixture of BNPs and MTHPA.

It is worth noting that there was a clear absorbance peak at 1712 cm^−1^ and 1690 cm^−1^, which also gradually disappeared up to 15 µm away from the HydBo layer. The absorbance peak at 1712 cm^−1^ was also observed in ATR-IR measurements when pure MTHPA hardener was mixed with BNPs, whereas the mixture of pure DGEBA and BNPs did not result in the emergence of any additional peaks (see Appendix E, Figure A8). The shift of absorption frequencies to 1712 cm^−1^ and 1,690 cm^−1^ can be attributed to the stretch of carbonyl bonds due to hydrogen bridging or chemical bonding to the hydroxyl groups of boehmite Another possible reason could be the water molecules, absorbed on the surface of HydBo layer or trapped inside the layered structure of crystals: the hydrolysis of anhydride results in the formation of hydroxyl-anhydrides that in turn result in a side reaction pathway of carboxyl-epoxy polyesterification [47]. The hydroxyl anhydride can further result in hydrogen-bonding that may explain the absorbance band at 1,690 cm^−1^.

## 4. Discussion

The soft interphase with 100–200 nm thickness in the outer region of HydBo is attributed to the soft interphase forms surrounding the particles in epoxy-BNP nanocomposites, as shown in our previous studies [16]. The chemical composition of the soft interphase is different from the bulk epoxy due to the existence of excess anhydride hardener that did not participate in any reaction, as observed in the AFM-IR spectrum taken from this region. The formation of an additional absorption band (1,710 cm^−1^) at the interphase, which is possibly related to species of anhydride with intra- or inter-molecular H-bonding, also confirms the altered chemistry of the epoxy in this region. Moreover, in the previous studies, the mechanical properties of bulk epoxy including the stiffness, energy dissipation, and cross-linking density were also affected by BNPs [16,17]. Here, measurements on a layered model system showed the chemical alteration of bulk epoxy. The AFM-IR results showing the preferential absorption of anhydride hardener and altered chemistry of the bulk support the hypotheses of distortion of stoichiometric ratio in the bulk. Therefore, further studies need to assess the effect of altered epoxy-hardener ratio on the bulk properties, such as stiffness, chemical structure, crosslink density, and energy dissipation, and correlate them with the presented results.

Although the location of a potential gradient and chemical alteration coincide, there is insufficient proof to conclude that the chemical alteration is the cause of potential gradient in the bulk epoxy. Therefore, other possible mechanisms for such long-range potential gradients must be considered. One possible mechanism is the transfer of charges (single ions or ions on particles or charge particle) from the HydBo into the epoxy layer. At the interface of HydBo and EP2, sharp needle-like nanostructures of boehmite grown up perpendicular to the EP2 plane, possibly enhance the injection and transfer of the trapped electrons to the bulk epoxy. Another consideration for the long-range potential gradient is the change in number of traps and localized energy states of the bulk epoxy. As an effect of disturbed stoichiometric-ratio and altered curing chemistry in the vicinity of boehmite, the resulting polymer network may have less traps for energy, which results in lower values of surface potential than for the bulk epoxy. To investigate the effect of trapping states and the mechanism of charge transfer, surface potential measurements must be carried out in an open-loop fashion with no voltage feedback, where the DC bias of the tip can be varied. In this case, where tip and sample come into contact at a point, electrons or holes can be injected and the change of the potential values will provide information about the charge traps [48].

## 5. Conclusions

The interaction between boehmite and epoxy was investigated successfully by designing a layered epoxy/boehmite/epoxy model sample. The hydrothermally synthesized boehmite coats the first epoxy layer (substrate). The outer morphology of the hydrothermally synthesized boehmite is in the form of nanoplatelets and nanoneedles perpendicular to the plane. This outer region comes in contact with the mixture of the resin and the hardener (second epoxy layer) while curing and thus, the formation of interphases is expected to be observed in this region. Intermodulation AFM provided maps of stiffness, electrostatic, and van der Waals forces. The contrast in the stiffness map revealed the formation of a soft phase with 100–200 nm thickness between the outer region of the boehmite layer and the epoxy. The soft interphase was hypothesized to be caused by preferential absorption of unreacted anhydride hardener molecules on the surface of boehmite. This hypothesis was finally proven by AFM-IR showing the correlated absorption peaks. In natural and man-made composites, synergistic material properties combining high-stiffness, strength, and toughness are often attributed to the very complex role of the soft interface and to a hierarchical structure based on mechanically inferior H-bonds [49,50,51]. Such a soft interphase often acts as an advanced binder, allowing for the energy dissipation and suppressing catastrophic crack propagation, thus promoting the toughness of the epoxy nanocomposite, as previously reported [9].

Using scanning Kelvin probe microscopy, a long-range interphase with a potential gradient width of over 10 µm was detected. Based on SEM-EDS measurements, boehmite particles did not diffuse far into the bulk epoxy and thus this effect does not play a role in the long-range potential gradient in the bulk epoxy. However, the distribution of carbon in the elemental analysis might indicate that polymer molecules penetrated in the boehmite layer, and thus we hypothesized that as a result of a selective interaction between the boehmite surface and epoxy components, the bulk polymer network is chemically altered, and this may give rise to the long-range potential interphase. AFM-IR investigations demonstrated the formation of a new absorption band at 1,710 cm^−1^ at the interface, up to a few microns away inthe bulk epoxy. The location of this chemical alteration coincides with the electrical interphase, demonstrating the long-range interaction and bulk effect of the presence of boehmite. Further studies are required to understand other possible mechanisms, such as bulk electrical formation and the consequence of preferential absorption of the hardener, which is hypothesized to alter the local stoichiometric balance of epoxy-anhydride systems. The presented model system and techniques open possibilities to investigate the interfacial properties of different kinds of polymer and inorganic interphases and study the effect of different surface modifications on the interphase properties.

## Figures and Tables

**Figure A1:**
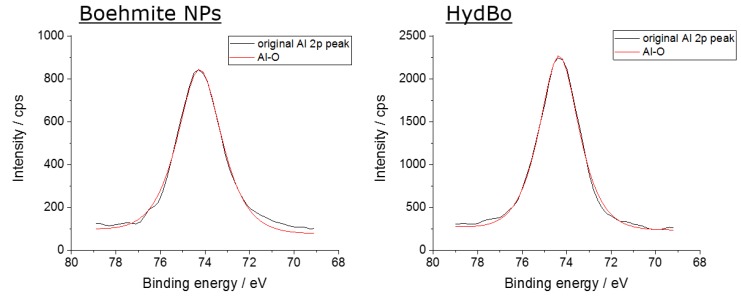
Comparison of Al p2 peak of commercial boehmite nanoparticles NPs (left) and hydrothermally synthesized boehmite layer on epoxy substrate (right).

**Figure A2:**
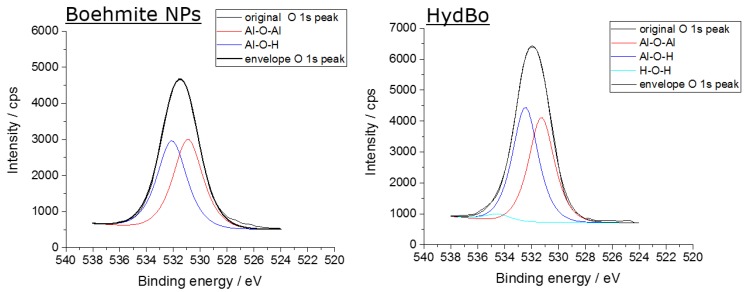
Comparison of O 1’s peak of commercial boehmite NPs (left) and hydrothermally synthesized boehmite layer on epoxy substrate (right).

**Figure A3:**
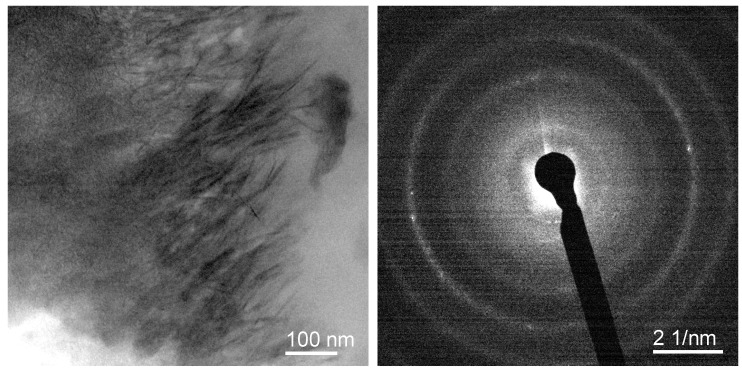
Bright field (BF) TEM micrograph of the outer region of HydBo layer in EP2 medium (left) diffraction pattern showing crystallinity of region in on the left (right).

**Figure A4:**
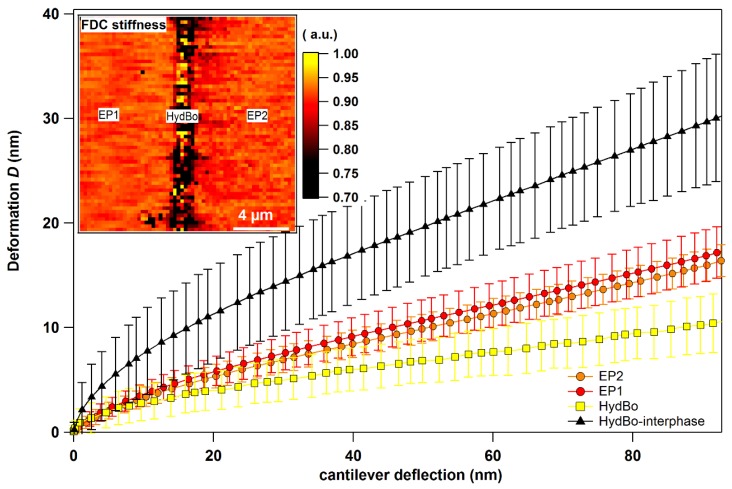
Force distance curves (FDC) stiffness map (inset image) and average force-distance curves of the cross-sectional surface of EP1/HydBo/EP2.

**Figure A5:**
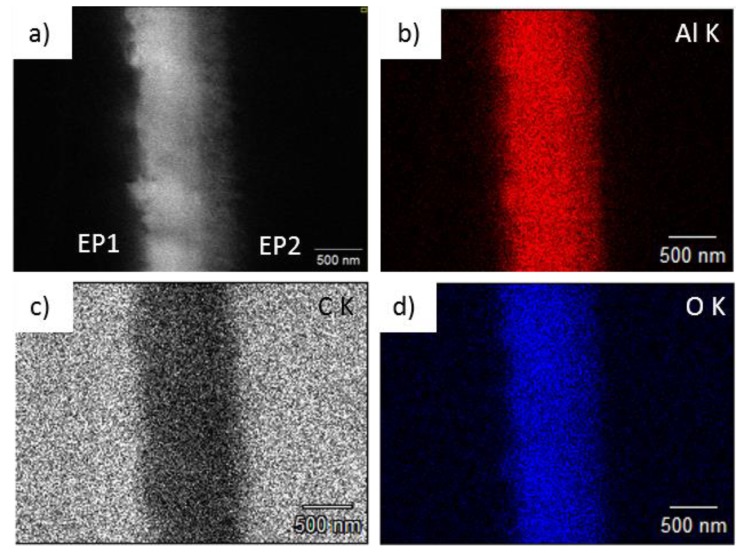
(**a**) 20 kV SEM micrograph of the microtome-slice EP1 (left)/HydBo/EP2 (right) sample and the corresponding net X-ray intensities of (**b**) aluminum (Al K), (**c**) carbon (C K) and (**d**) oxygen (O K) extracted from an 20 kV EDS hypermap of 256 × 192 pixels after acquisition of 67 frames @ 4.4 s/frame.

**Figure A6:**
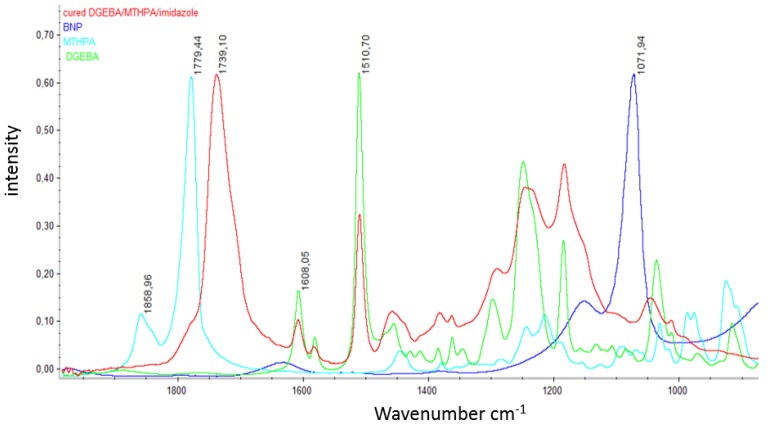
Room temperature ATR-IR spectra of BNPs, epoxy resin DGEBA, anhydride hardener MTHPA, and the thermally-cured DGEBA/MHTPA/imidazole(accelerator) with 100:90:1 mixing ratio and 120 curing temperature.

**Figure A7:**
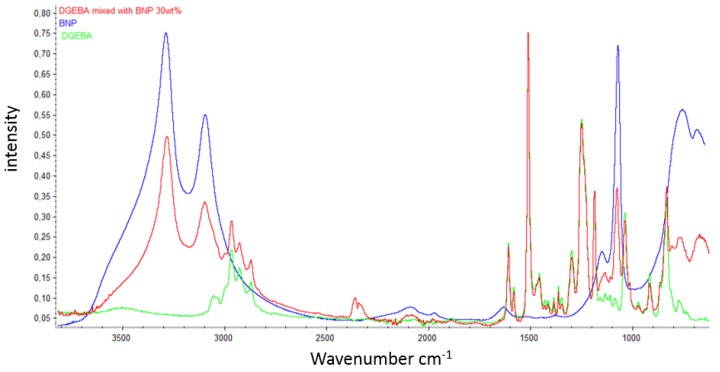
Room temperature ATR-IR spectra of BNPs, epoxy resin DGEBA and the masterbatch mixture of BNPs and DGEBA.

## Appendix A

We verified the chemical structure of HydBo with XPS and compared it with commercially available boehmite nanoparticles (Disperal HP14, Sasol, Germany). During the XPS measurement, the sample is irradiated with X-rays, enabling the removal of near-core electrons from existing atoms. These electrons will be detected and are characteristic of the corresponding atoms. XPS is a surface sensitive method due to working under ultra-high vacuum, where the main information depth is about 5–7 nm. As seen in Figure A1, the Al 2p peaks show no significant difference. The binding energy of commercially available boehmite NP is 74.3 eV, and 74.4 eV for HydBo. According to previous studies, O 1’s spectra provide more information about the crystal structure, hydroxyl groups, and absorbed water of boehmite [52]. When comparing the O 1’s peaks of the purchased and self-produced HydBo, it becomes clear that both are very similar. The O 1’s peak was fitted in both cases. Signals were found for the Al-O-Al and the Al-OH bond at 532.2 and 531.0 eV for the commercially available boehmite. In the HydBo, the binding energies were shifted by 0.2 eV to higher binding energies. The percentage ratios of Al-O-Al to Al-OH for both boehmite types are shown in Table A1. The percentage contents of the HydBo are minimally lower because the sample still contains residual water from the production process.

**Table A1:** Comparison of the percentage ratios of the fitted O 1’s peaks.

Sample	Al-O-Al in %	Al-O-H in %	H_2_O
Commercially available boehmite (BNP)	49.7	50.3	-
HydBo	48.2	49.7	2.1

## Appendix B

An ultramicrotome cut of EP1/HydBo/EP2 with 100 nm nominal thickness was examined using a 2200 FS (JEOL, Japan) transmission electron microscope (TEM) working at an acceleration voltage of 200 keV. The morphology of the outer shell of HydBo (Figure A3, left) is consistent with SEM micrographs (Figure 2), demonstrating the needle- and platelet-like structures with low crystallinity (Figure A3, right).

## Appendix C

Conventional AFM (force distance curves, FDC) was carried out on the cross-sectional surface of EP1/HYdBo/EP2 using a silicon probe with a spring constant of 50 nN/nm, and resonance frequency of 329.43 kHz (Nanosensors, Switzerland). The average force-distance curves (repulsive regime) and the FDC stiffness map are presented in Figure A4. A comparison of force-distance curves from EP1 and EP2 in bulk (away from interphase) shows that the Young’s moduli of epoxies on both sides are similar. The FDC map, taken from different distances from the boehmite layer, does not show any significant changes in the stiffness values. Thus, it can be concluded that despite having a long-range (10 µm) chemical interphase between HydBo and epoxy, the mechanical interphase is not observable in this range.

## Appendix E

Attenuated total reflection infrared (ATR/IR) spectra were collected using an FT-IR spectrometer (Smart Orbit; Thermo Fischer Scientific; Karlsruhe, Germany) at room temperature. Figure A6 shows the IR spectra of anhydride-cured epoxy as well as individual components of the epoxy including pure resin and anhydride hardener, as well as pure BNP (powder). The most pronounced peak results from the reaction between oxirane groups of DGEBA with MTHPA and the formation of an ester band at 1739 cm^−1^.

The mixture of DGEBA with BNP via three-roll milling is examined with ATR, whereas no specific change in the spectrum regarding any possible reaction was found (Figure A7). However, evaluating the IR spectrum of the mixture of MTHPA with BNPs prepared by three-roll milling (Figure A8) shows that boehmite and anhydride react even at room temperature and thus an additional absorption at 1710 cm^−1^ regarding either a chemical bond or an H-bond appears after mixing. This information confirms that boehmite interacts more preferentially with an anhydride component than with the DGEBA resin.
